# Study on the Mechanism of Qing-Fei-Shen-Shi Decoction on Asthma Based on Integrated 16S rRNA Sequencing and Untargeted Metabolomics

**DOI:** 10.1155/2023/1456844

**Published:** 2023-02-15

**Authors:** Haibo Hu, Guojing Zhao, Kun Wang, Ping Han, Haiyan Ye, Fengchan Wang, Na Liu, Peixia Zhou, Xuechao Lu, Zhaoshan Zhou, Huantian Cui

**Affiliations:** ^1^Qingdao Traditional Chinese Medicine Hospital (Qingdao Hiser Hospital), Qingdao University, Qingdao, China; ^2^Shandong Provincial Key Laboratory of Animal Cell and Developmental Biology, School of Life Sciences, Shandong University, Jinan, Shandong, China

## Abstract

Qing-Fei-Shen-Shi decoction (QFSS) consists of *Prunus armeniaca* L., *Gypsum Fibrosum*, *Smilax glabra Roxb.*, *Coix lacryma-jobi* L., *Benincasa hispida (Thunb.) Cogn.*, *Plantago asiatica* L., *Pyrrosia lingua (Thunb.) Farw.*, *Houttuynia cordata Thunb.*, *Fritillaria thunbergii Miq.*, *Cicadae Periostracum*, and *Glycyrrhizae Radix Et Rhizoma Praeparata Cum Melle*. QFSS shows significant clinical efficacy in the treatment of asthma. However, the specific mechanism of QFSS on asthma remains unclear. Recently, multiomics techniques are widely used in elucidating the mechanisms of Chinese herbal formulas. The use of multiomics techniques can better illuminate the multicomponents and multitargets of Chinese herbal formulas. In this study, ovalbumin (OVA) was first employed to induce an asthmatic mouse model, followed by a gavage of QFSS. First, we evaluated the therapeutic effects of QFSS on the asthmatic model mice. Second, we investigated the mechanism of QFSS in treating asthma by using an integrated 16S rRNA sequencing technology and untargeted metabolomics. Our results showed that QFSS treatment ameliorated asthma in mice. In addition, QFSS treatment affected the relative abundances of gut microbiota including *Lactobacillus*, *Dubosiella*, *Lachnospiraceae_NK4A136_group,* and *Helicobacter*. Untargeted metabolomics results showed that QFSS treatment regulated the metabolites such as 2-(acetylamino)-3-[4-(acetylamino) phenyl] acrylic acid, D-raffinose, LysoPC (15 : 1), methyl 10-undecenoate, PE (18 : 1/20 : 4), and D-glucose6-phosphate. These metabolites are associated with arginine and proline metabolism, arginine biosynthesis, pyrimidine metabolism, and glycerophospholipid metabolism. Correlation analysis indicated that arginine and proline metabolism and pyrimidine metabolism metabolic pathways were identified as the common metabolic pathways of 16s rRNA sequencing and untargeted metabolomics. In conclusion, our results showed that QFSS could ameliorate asthma in mice. The possible mechanism of QFSS on asthma may be associated with regulating the gut microbiota and arginine and proline metabolism and pyrimidine metabolism. Our study may be useful for researchers to study the integrative mechanisms of Chinese herbal formulas based on modulating gut microbiota and metabolism.

## 1. Introduction

Asthma is a global common chronic inflammatory airway disease, which is characterized by increased mucus secretion in the airways mediated by multiple cells, as well as inflammatory factors, airflow obstruction, and airway remodeling. The clinical symptoms usually manifest as wheezing, coughing, and dyspnea. Globally, approximately 300 million people suffer from asthma [1]. In China, epidemiological surveys have shown that the overall prevalence of asthma in people over 20 years of age is 4.2% [2]. Asthma treatment regimens mainly include bronchodilators (such as beta-2 agonists and aminophylline), antiallergic inflammatory drugs (such as glucocorticoids and sodium cromoglicate), and immunomodulators. However, most therapeutic drugs have certain side effects, such as nausea and vomiting, when intravenous theophylline is used [3] and cardiovascular side effects when *β*-adrenergic agonists [4] are used. In addition to the potential side effects, the antiasthmatic medications in current clinical treatments also impose significant financial strain on patients. Therefore, the development of safe and effective medicines to relieve asthma has become a lucrative research topic.

Traditional Chinese medicine (TCM) has accumulated thousands of years of clinical experience in the treatment of asthma. A clinical randomized multicenter trial found that Ping-Chuan-Yi-Qi (PCYQ) granules significantly improved peak expiratory flow rate (PEFR) and reduced serum cytokine levels in asthmatic patients [5]. A meta-analysis showed that the combination of TCM with conventional treatment improved the clinical symptoms of asthma patients with a better safety profile compared to the conventional treatments [6]. Wang et al. summarized the research progress of TCM in the treatment of asthma and discussed in detail the studies of extremely effective components of TCM in regulating immune imbalance in asthma patients [7]. Elucidating the mechanism of TCM for asthma can help the development of new antiasthmatic drugs and promote the modernization of TCM at the same time.

Gut microbiota is a general term for the microbiota that colonize in the intestines. Gut microbiota are diverse, and mainly include *Firmicutes*, *Bacteroides*, *Proteobacteria*, and *Actinomycetota,* and each phylum is distributed in different parts of the intestine in different proportions. These bacteria play an important role in maintaining the dynamic equilibrium between the internal and external environments. The concept of the “*gut-lung axis*” emphasizes the interaction between microorganisms indicated by the epithelium of the gastrointestinal and respiratory tracts [8]. Recent studies have shown that gut microbiota play a key role in the pathogenesis of asthma [9]. *Bifidobacteria, Akkermansia,* and *Faecalibacterium* are less abundant and *Candida* and *Rhodotorula* are more abundant in the gut of children with asthma compared to that in normal subjects, and this dysbiosis may further aggravate the immune imbalance [10]. In addition, the long-term use of hormones and antibiotics in asthma patients can also aggravate gut dysbiosis, which is not conducive to the recovery of the disease [11]. Regulating gut microbiota may provide new therapeutic ideas for treating asthma. Recuperating lung decoction increases the levels of beneficial bacteria such as *Lactobacillus* and *Bifidobacterium* [12]. Gu-Ben-Fang-Xiao decoction can promote T regulatory cell (Treg) differentiation and thus relieve asthma by regulating gut microbiota [13]. Tuo-Min-Ding-Chuan decoction can affect phenylalanine, ascorbate, and aldarate metabolism by regulating the abundance of *Desulfovibrio*, *Butyricimonas*, *Prevotella*, and other microbiota in the gastrointestinal tract, and glutathione metabolism to treat asthma [14].

Qing-Fei-Shen-Shi decoction (QFSS), which consists of *Prunus armeniaca* L., *Gypsum Fibrosum*, *Smilax glabra Roxb.*, *Coix lacryma-jobi* L., *Benincasa hispida (Thunb.) Cogn.*, *Plantago asiatica* L., *Pyrrosia lingua (Thunb.) Farw.*, *Houttuynia cordata Thunb.*, *Fritillaria thunbergii Miq.*, *Cicadae Periostracum*, and *Glycyrrhizae Radix Et Rhizoma Praeparata Cum Melle*, has been used for the treatment of acute attack asthma in the clinic for more than 30 years. A clinical study showed that QFSS significantly improved respiratory function and reduced the frequency of attacks in patients with asthma [15, 16]. However, the specific mechanism of QFSS in the treatment of asthma remains unclear. In this study, ovalbumin (OVA) was first employed to induce an asthmatic mouse model, followed by a gavage of QFSS. First, we evaluated the therapeutic effects of QFSS on asthmatic mice. Second, we investigated the mechanism of QFSS in treating asthma by integrating 16s rRNA sequencing technology and untargeted metabolomics.

## 2. Materials and Methods

### 2.1. Reagents

The detailed information of reagents has been presented in supplementary materials.

### 2.2. Preparation and Quality Control of QFSS

Briefly, 12 g of *Prunus armeniaca* L., 30 g of *Gypsum Fibrosum*, 15 g of *Smilax glabra Roxb.*, 30 g of *Coix lacryma-jobi* L., 30 g of *Benincasa hispida (Thunb.) Cogn.*, 15 g of *Plantago asiatica* L., 15 g of *Pyrrosia lingua (Thunb.) Farw.*, 30 g of *Houttuynia cordata Thunb.*, 12 g of *Fritillaria thunbergii Miq.*, 12 g of *Cicadae Periostracum*, and 9 g of *Glycyrrhizae Radix Et Rhizoma Praeparata Cum Melle* were weighed and mixed. Then, water was added so that the volume ratio of herbs to water was 1:8. Then, the herbs were soaked for 30 min, and decocted twice for 60 min each time. The water extracts of QFSS were then concentrated to 5.4 g crude herb/mL.

### 2.3. Experimental Animals

Sixty adult male specifically pathogen-free (SPF) grade BALB/c mice weighing 20 g ± 2 g were provided by Beijing Huafukang Co. Ltd., with Certificate of Approval No. being SCXK (Beijing) 2019-0008. The mice were housed in a SPF-grade clean environment, with 5 mice per cage at a temperature of 22°C ± 2°C, a humidity of 50% ± 15%, and a day/night time of 12 h/12 h, and free access to food and water was provided. All animal experiments were performed in accordance with the National Institutes of Health (NIH) guidelines for the care and use of laboratory animals, and all procedures were approved by the Animal Medicine and Animal Protection Ethics Committee of Qingdao University (approval no. QDU-AEC-202282).

### 2.4. Induction of Asthma in Mice

After 1 week of acclimatization feeding, mice were intraperitoneally injected with 0.2 mL of OVA and aluminum hydroxide (alum) mixture (40 *μ*g OVA + 1 mg of 10% alum + 200 *μ*L of saline) on days 0, 7, and 14. Starting from day 21, mice were placed in an airtight chamber (20 × 30 × 15 cm) and received nebulized inhalation of 2% OVA for 30 min, once every 2 days for 4 consecutive weeks [17].

### 2.5. Grouping and Dosing Regimen

**Figure 1:**
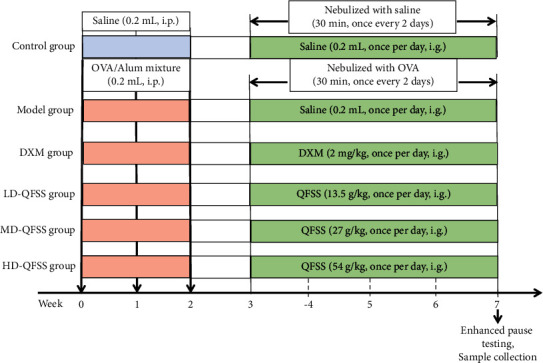
Experimental design of this study.

The 60 mice were randomly divided into the control group, model group, DXM group, LD-QFSS group, MD-QFSS group, and the HD-QFSS group. After 1 week of acclimatization feeding, mice from model, DXM, LD-QFSS, MD-QFSS, and HD-QFSS groups received OVA treatment to induce asthma. Meanwhile, mice in the control group were intraperitoneally injected with 0.2 mL saline on days 0, 7, and 14, and received nebulized inhalation of saline for 30 min, once every 2 days for 4 consecutive weeks starting from day 21. Starting from day 21, mice in the control and model groups were given 0.2 mL saline once per day via intragastric administration. The mice in the DXM group, LD-QFSS group, MD-QFSS group, and the HD-QFSS group were given DXM 2 mg/kg [18], QFSS 13.5 g/kg, QFSS 27 g/kg, and QFSS 54 g/kg once per day for 4 weeks was administered via intragastric administration, respectively (Figure 1). Clinically, the dosage of QFSS for adult patients (body weight: 70 kg) was 210 g (the total raw materials) once per day. The dosage for mice was calculated by following the classical pharmacological formula. MD-QFSS (human equivalent dose) = 210 g (the total raw materials)/70 kg (human weight) × 9 (conversion coefficient). The low dose and high dose were 0.5× and 2× of the middle dose, respectively.

### 2.6. Enhanced Pause (Penh) Testing

After 4 weeks of QFSS intervention, mice were anesthetized with 1% sodium pentobarbital. The trachea was fully exposed, and a catheter was inserted after the condition of the mice was stable. One end of the catheter was connected to the trachea and the other end was connected to the EMKA Small Animal Lung Function Test System Ventilator. The mice in the control group were first exposed to phosphate-buffered saline (PBS) nebulizer to obtain Penh baseline values, by direct administration of acetylcholine aerosol via the ventilator. Each mouse was then exposed to different concentrations of acetylcholine (6.25, 12.5, 25, and 50 g/L) and Penh values were recorded for 3 min at each dose.

### 2.7. Serum and Bronchoalveolar Lavage Fluid (BALF) Collection

After 4 weeks of QFSS intervention, mice were anesthetized and blood was collected from the abdominal aorta using a syringe, and the collected blood was centrifuged at 3,000 rpm/min for 15 min to collect the serum.

Then, the mice were sacrificed, and the thoracic cavity was opened. The cervical trachea was exposed layer by layer, and a Lanz incision was performed on the trachea. The mouse lavage needle was inserted into the lower end of the right main bronchus, and the trachea and mouse lavage needle were ligated by surgical sutures. Then, the hilum of the left lung was firmly ligated using surgical sutures to ensure that the left lung was in an airtight position. Using a syringe, 1 mL of saline was withdrawn and connected to the lavage needle ligated to the cervical trachea, where saline was slowly injected into the right lung of mice. Then, the saline was gently withdrawn back to obtain BALF after staying in the alveoli for 15–30 s. Saline was closely observed for exudation during the lavage. This injection and withdraw procedures were repeated 3 times, and a total of approximately 2.5 mL of BALF was recovered from each mouse.

### 2.8. Lung Wet-to-Dry (W/D) Weight Ratio

The left lung of the mouse that was not lavaged was retained. The wet mass (W) of the left lung was measured, followed by drying in a constant temperature oven at 80°C for 48 h until the lung weight no longer decreased. At this point, the lung was weighed as the dry weight (D), and the lung W/D ratio was calculated.

### 2.9. BALF Total Cell Count, Eosinophil Count, and the Total Protein Concentration Assay

The BALF was centrifuged at 4°C for 10 min at 3,000 rpm/min, and the cell precipitate and supernatant were collected separately. The total number of cells in the BALF precipitate was enumerated using a cell counting plate. The eosinophil count in BALF was enumerated using Wright's-Giemsa staining. The total protein concentration in BALF supernatant was measured using the bicinchoninic acid (BCA) total protein concentration assay kit.

### 2.10. Pathological Staining of Lung Tissues

After 24 h of final stimulation, the mice were sacrificed and the lung tissues of mice from each group were collected, followed by fixation in the formalin solution. Then, the fixed tissues were embedded in paraffin, cut into 3 *μ*m sections, and routinely stained with hematoxylin and eosin (HE) and Masson. The histopathological changes of the lung tissues of each group were observed under a light microscope, and the inflammation levels of the lung tissues in HE staining were scored based on the previous study [19]. The Masson staining was also scored as described previously [19].

### 2.11. ELISA

The levels of immunoglobulin E (IgE) in serum and interleukin (IL)-4, IL-5, IL-13, and interferon-gamma (IFN-*γ*) in BALF supernatants were measured based on the ELISA kit instructions.

### 2.12. Lung Tissue Biochemical Tests

50 mg of lung tissues were weighed and 450 *μ*L of normal saline was added. After that, the solution underwent ultrasonic homogenization before centrifugation at 4°C for 10 min at 3,000 rpm/min. Lung tissue homogenates were prepared by collecting the supernatants of homogenized tissues. The BCA kit was used to normalize the protein levels in tissue homogenates, which were then used to detect the activities of superoxide dismutase (SOD), glutathione peroxidase (GSH-Px), and malondialdehyde (MDA) levels according to the instructions of the kit.

### 2.13. 16S rRNA Sequencing

#### 2.13.1. Fecal Specimen Collection, Genomic DNA Extraction, and Storage

Six animals were randomly selected in control, model, and HD-QFSS groups for the 16S rRNA sequencing analysis. After the mice were anesthetized and sacrificed, the cecum was isolated by opening the abdominal cavity, and the contents of the end of the cecum were obtained and placed in sterile Eppendorf (EP) tubes and stored instantly in a low-temperature freezer at −80°C. Under aseptic conditions, 200 mg of fecal samples were weighed and added to 2 mL EP tubes, and genomic DNA was extracted from each sample according to the operating instructions of the Fecal DNA Extraction Kit. The quality and concentration of the extracted genomic DNA were measured using a Thermo Nanodrop 2000 spectrophotometer and 1% agarose gel. DNA was diluted to 1 ng/*µ*L with sterile water, depending on the concentration.

#### 2.13.2. Polymerase Chain Reaction (PCR) Amplification and Sequencing of 16S rRNA Gene

Based on the characteristics of the amplified 16S region, PCR amplification of the hypervariable region of 16S rDNAV3-V4 was performed with the amplification primers 338F (5′-ACTCCTACGGGAGGCAGCAG-3′) and 806R (5′-GGACTACHVGGGTWTCTAAT-3′). After performing 2% agarose gel electrophoresis to quantify the amplified products, the Illumina NovaSeq platform was selected for sequencing, and a 250 bp paired-end sequence was obtained. After the raw data were obtained by sequencing, the raw data were assembled and filtered to obtain the clean data. Then, the entire clean data of all samples were clustered using UParse software (UParsev7.0.1001) and the sequences were clustered into operational taxonomic units (OTUs) with 97% agreement.

#### 2.13.3. Sequencing Data Processing and Analysis

Diversity analysis was performed on the sequencing data results. Shannon and Simpon indices were used to assess the gut microbiota alpha diversity of each group. The results of beta diversity analysis were presented by principal coordinate analysis plot (PCoA) analysis, an unconstrained data downscaling analysis that presents similarities and differences in community composition across sample groups. The Wilcoxon rank-sum test was used to test for differences between groups in diversity indices. In addition, OTU was used for species classification of OTU by comparing it to the database, and the species abundance of each group of samples was analyzed at the phylum level and genus level. Finally, the analysis of the Phylogenetic Investigation of Communities by Reconstruction of Unobserved States database (PICRUSt) was used to predict the relevant gene pathways that may be affected by each group of differential microbiotas.

#### 2.13.4. Metabolomics Analysis

Six animals were randomly selected in control, model, and HD-QFSS groups for the untargeted metabolomics analysis. A 100 mg of lung tissue was added to 500 *μ*L of 80% methanol solution, vortexed and shaken, and left to stand in an ice bath for 5 min. The tissue was centrifuged at 15,000*g* and 4°C for 20 min. Then, the supernatant was collected and diluted with water to obtain a 53% methanol level. After that, the supernatant was collected and centrifuged for 20 min at 15,000*g* and 4°C to obtain a tissue homogenate for untargeted metabolomic analysis using liquid chromatography-mass spectrometry (LC-MS). All samples were obtained in equal amounts individually and mixed; these were used as the quality control (QC) samples. The specific chromatographic and mass spectrometric conditions and data processing and analysis were performed according to our previously published article [20, 21] (supplementary material (available here)). Spearman's correlation analysis was used to find the correlation between therapeutic indicators, differential metabolites, and changed gut microbiota.

#### 2.13.5. Statistical Processing

Statistics and analysis were performed using SPSS 20.0 statistical software, and data were expressed as mean ± standard deviation. One-way analysis of variance (ANOVA) followed by Tukey's post-hoc test was used for comparison among groups. A difference of *P* < 0.05 was considered to be statistically significant.

## 3. Results

### 3.1. Therapeutic Effects of QFSS on the Asthma Model Mice

The Penh values in the model group were significantly higher than those in the control group after administering interventions of different concentrations of Ach (*P* < 0.01, respectively). Compared to the model group, Penh values were significantly lower in the DXM and HD-QFSS groups after 6.25 mg/mL Ach intervention (*P* < 0.01 and *P* < 0.05, respectively); Penh values significantly decreased in the DXM, MD-QFSS, and HD-QFSS groups after the 12.5 mg/mL, 25 mg/mL, and 50 mg/mL Ach interventions (all *P* < 0.01) (Table 1).

**Figure 2:**
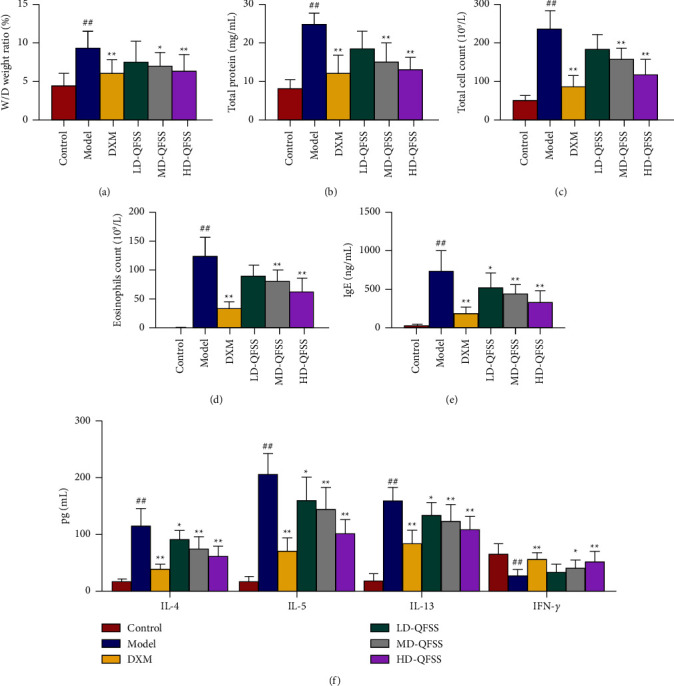
QFSS treatment ameliorated asthma in mice. (a–c) QFSS treatment decreased the W/D ratio of lung tissue (a), total protein concentration (b), and total cell count (c), in BALF in asthmatic model mice (d). The numbers of eosinophils in BALF were observed by Wright's-Giemsa staining. Mice which received QFSS showed lower eosinophils count in BALF than with mice in the model group. (e) Serum IgE level was tested by ELISA. QFSS treatment reduced the IgE level in mice with asthma. (f) Cytokine levels of IL-4, IL-5, IL-13, and IFN-*γ* in BALF were investigated using ELISA. QFSS treatment decreased the levels of IL-4, IL-5, and IL-13, and increased the IFN-*γ* level in BALF in mice with asthma. Control, model, DXM, LD-QFSS, MD-QFSS, and HD-QFSS groups (*n* = 10 per group). ^##^*P* < 0.01 compared with the control group; ^*∗*^*P* < 0.05 compared with the model group; ^*∗∗*^*P* < 0.01 compared with the model group.

The lung tissue W/D ratio was significantly higher in asthmatic mice compared to that in the control group (*P* < 0.01). The ratios were significantly lower in the DXM, MD-QFSS, and HD-QFSS treatment groups compared to that in the model group (*P* < 0.01, *P* < 0.05, and *P* < 0.01, respectively; Figure 2(a)). The total protein content of BALF supernatant was significantly increased in the model group compared to that in the control group (*P* < 0.01), and significantly decreased in the DXM, MD-QFSS, and HD-QFSS groups compared to that in the model group (all *P* < 0.01; Figure 2(b)). The total cell count in BALF was significantly increased in the model group compared to that in the control group (*P* < 0.01), and the DXM, MD-QFSS, and HD-QFSS groups significantly decreased the total cell count in BALF compared to the model group (all *P* < 0.01; Figure 2(c)). More eosinophils were found in the BALF of the model group compared to that in the control group (*P* < 0.01), and lower eosinophil counts were found in the BALF of the DXM, MD-QFSS, and HD-QFSS groups compared to that in the model group (all *P* < 0.01; Figure 2(d)). In addition, serum IgE levels were elevated in the model group compared to those in the control group (*P* < 0.01), but serum IgE levels were significantly lower in the DXM, LD-QFSS, MD-QFSS, and HD-QFSS groups compared to those in the model group (*P* < 0.01, *P* < 0.05, *P* < 0.01, and *P* < 0.01, respectively; Figure 2(e)).

**Figure 3:**
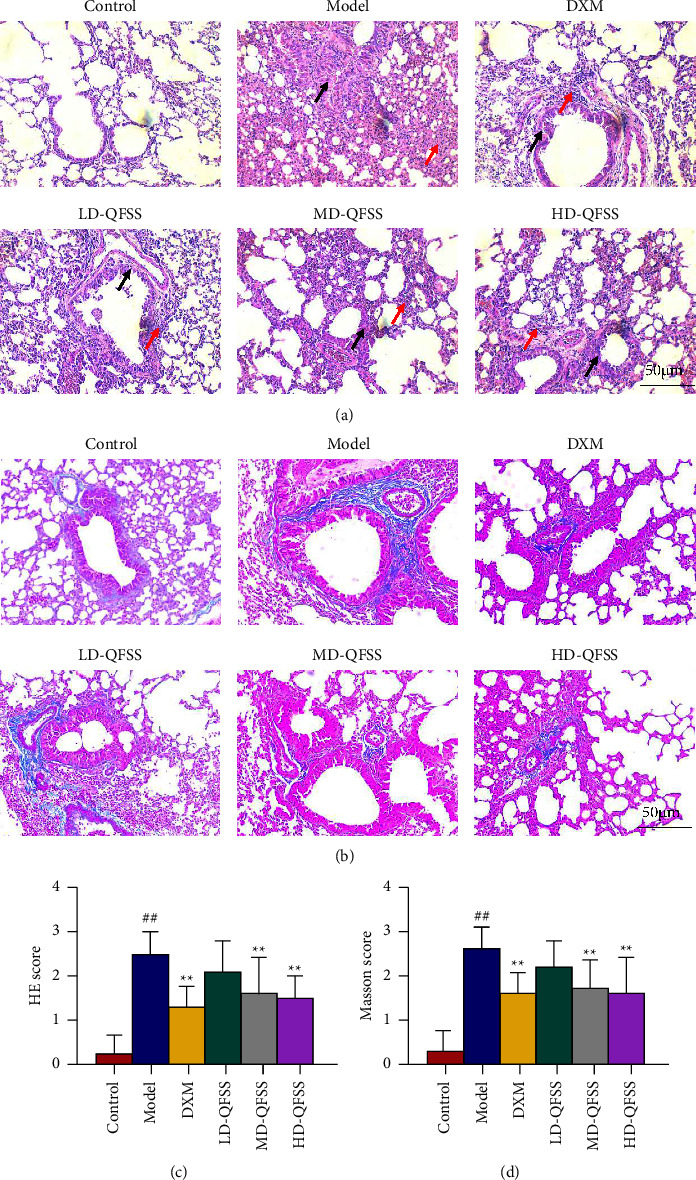
QFSS treatment improved the pathological changes in mice with asthma. (a, c) HE staining showed that QFSS treatment improved the pathological changes (disorderly bronchial epithelial cell arrangement, bronchial smooth muscle thickening, and a large number of inflammatory cells) in lung (a) and decreased the inflammation score (c). Black arrows indicated the impaired structure of bronchus and red arrows indicated the infiltration of proinflammatory cells (magnification: 200×). (b, d) Masson staining showed that QFSS reduced the deposition of collagen contents in lung (b) and decreased the Masson staining score (d) in asthmatic model mice (magnification: 200×).

HE staining showed no pathological changes in the lung tissue of the control group. Disorderly bronchial epithelial cell arrangement, bronchial smooth muscle thickening, and a large number of inflammatory cells could be observed in the model group, while DXM and QFSS treatment improved pathological changes in the lungs (Figure 3(a)). Similarly, inflammation scores were significantly higher in the model group than those of the control group (*P* < 0.01), and inflammation scores were lower in the DXM, MD-QFSS, and HD-QFSS groups than in the model group (*P* < 0.01, respectively; Figure 3(c)). Masson staining showed a significant increase in the collagen deposition in the lungs of the asthmatic model mice compared with mice in the control group. QFSS and DXM treatment reduced the collagen deposition in the lungs of mice infected with asthma (Figure 3(b)). Likewise, the Masson staining score was higher in the model group than that of the control group (*P* < 0.01), and the Masson scores were lower in the DXM, MD-QFSS, and HD-QFSS groups (*P* < 0.01, respectively; Figure 3(d)).

### 3.2. Effects of QFSS on Inflammation and Oxidative Stress in Asthmatic Mice

We measured cytokine levels in BALF to see if QFSS affected the level of inflammation in the asthma model mice. In addition, MDA levels, SOD, and GSH-Px activities were measured in lung tissue homogenates to assess the effect of QFSS on oxidative stress. ELISA results showed that the levels of IL-4, IL-5, and IL-13 were increased as expected in the model group compared to those in the control group (*P* < 0.01), while these cytokines were significantly reduced in the DXM group compared to those in the model group (*P* < 0.01, respectively). QFSS treatment reduced IL-4, IL-5, and IL-13 levels in a dose-dependent manner (Figure 2(f)). IFN-*γ* levels were significantly lower in the model group compared to those in the control group (*P* < 0.01). DXM, MD-QFSS, and HD-QFSS all increased IFN-*γ* levels compared to the model group (*P* < 0.01, *P* < 0.05, and *P* < 0.01, respectively; Figure 2(f)). Compared to the control group, SOD and GSH-Px activities decreased and MDA levels increased in the model group (all *P* < 0.01), whereas SOD and GSH-Px activities increased and MDA levels decreased in the QFSS high-dose group compared to those in the model group (*P* < 0.01, *P* < 0.05, and *P* < 0.01, respectively; Table 2).

The abovementioned results showed that the asthma model was established successfully, and that QFSS had a therapeutic effect on asthma, which was most significant at a high dose. Therefore, the HD-QFSS group was selected for the subsequent gut microbiota and lung metabolite study.

### 3.3. Effect of QFSS on Gut Microbiota of Asthmatic Mice

**Figure 4:**
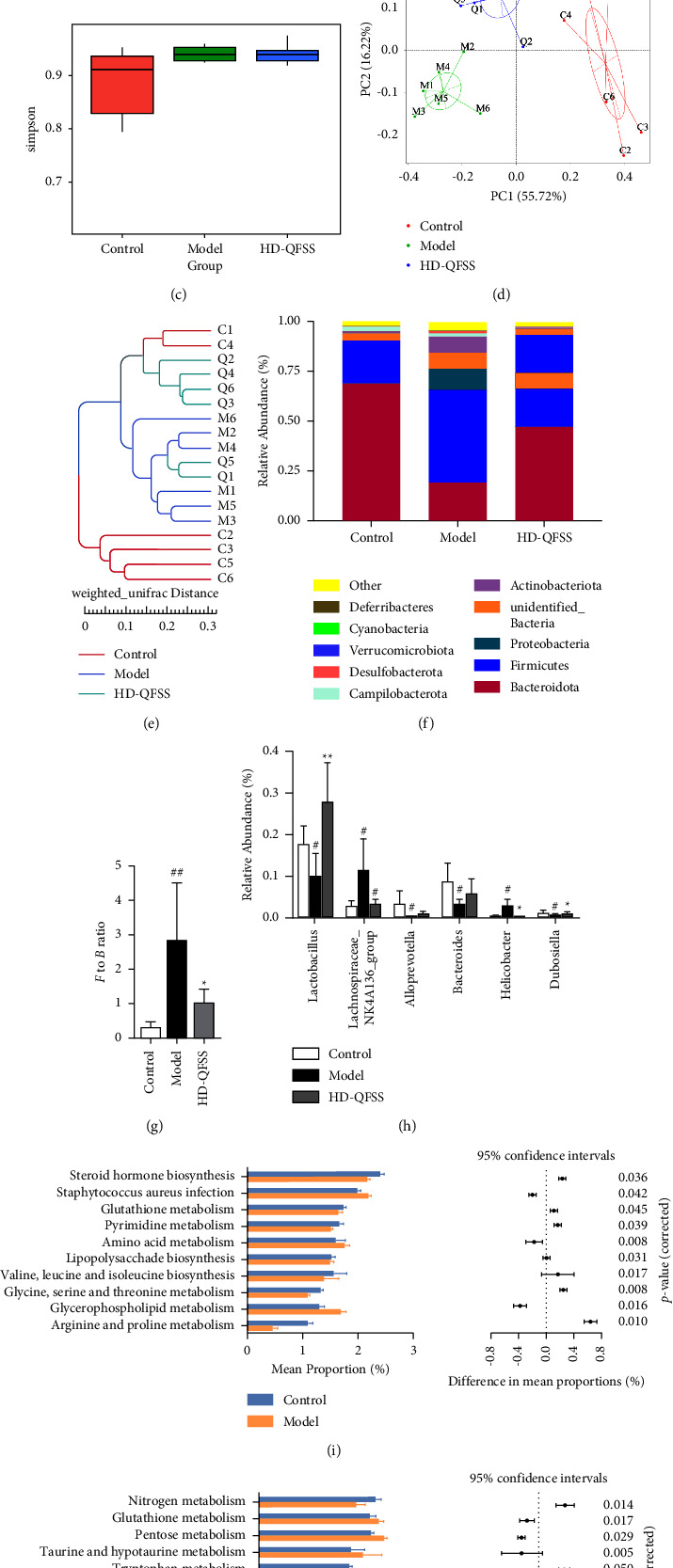
QFSS treatment affected the gut microbiota community in asthmatic mice. (a) Dilution curve of each sample showed that the depth of sequence was reasonable and appropriate. (b, c) Alpha diversity of gut microbiota in each group was assessed through Shannon (b) and Simpson (c) indexes. There were no significant differences in alpha diversity of gut microbiota in each group (d, e) PCoA (d) and system clustering tree (e) indicated more similar beta diversity between HD-QFSS and control groups than that between asthma and control groups (C: control group; M: model group; Q: HD-QFSS group). (f, g) At the phylum level, QFSS treatment decreased the F-to-B ratio in asthma model mice. (h) At the genus level, QFSS treatment affected the relative abundances of gut microbiota including *Lactobacillus*, *Dubosiella*, *Lachnospiraceae_NK4A136_group,* and *Helicobacter*. (i, j) Metabolic pathways were predicted using PICRUSt analysis based on the 16S rRNA sequencing data. The common pathways between control and model groups (i) and model and HD-QFSS groups (j) were marked in red. Control, model, and HD-QFSS groups (*n* = 6 per group).

To investigate the changes of QFSS on the gut microbiota of asthmatic mice, we analyzed the fecal microbiota of different groups of mice by using 16S rRNA high-throughput sequencing. The dilution curve in each sample tended to be flat after the number of sequences increased to 10,000. Few novel OTUs can be identified after the number of sequences increased to 40,000, indicating that the depth of sequencing is reasonable and appropriate (Figure 4(a)). The abundance and diversity of microbial communities within the samples were analyzed using alpha diversity (Shannon's index and Simpson's index). The results showed that there were no significant differences in the Shannon index and the Simpson index in each group (Figures 4(b) and 4(c)). Next, we analyzed the composition of microbial communities of different samples using beta diversity and evaluated them by PCoA and clustering analysis. The PCoA results showed that the sample points in the model group were completely separated from the control group, and the sample points in the HD-QFSS group were closer to those in the control group (Figure 4(d)). Clustering analysis also showed similar results (Figure 4(e)).

The composition of the gut microbiota in each group of samples at the phylum level is shown in Figure 4(f), with *Firmicutes* and *Bacteroidetes* as the dominant taxa. The *Firmicutes/Bacteroidetes* (F to B) ratio was significantly higher in the model group than that in the control group (*P* < 0.01), while the F-to-B ratio was significantly lower in the HD-QFSS group than that in the model group (*P* < 0.05, Figure 4(g)). At the genus level, the relative abundance of *Lachnospiraceae_NK4A136_group* (*P* < 0.05) and *Helicobacter* (*P* < 0.05) was significantly higher in the asthmatic mouse model than that in the control group, and the relative abundance of *Lactobacillus* (*P* < 0.05), *Alloprevotella* (*P* < 0.05), *Bacteroidota* (*P* < 0.05), and *Dubosiella* (*P* < 0.05) were significantly decreased. QFSS significantly increased the relative abundance of *Lactobacillus* (*P* < 0.01) and *Dubosiella* (*P* < 0.05), and decreased the relative abundance of *Lachnospiraceae_NK4A136_group* (*P* < 0.05) and *Helicobacter* (*P* < 0.05) compared to the model group (Figure 4(h)).

The functional changes of intestinal microbiota between the control and model groups and between the model and HD-QFSS groups were predicted by PICRUSt analysis. Differential metabolic pathways (*P* < 0.05) with the top ten proportions were listed in Figures 4(i) and 4(j). The common pathways (control vs. model and model vs. HD-QFSS) included glutathione metabolism, pyrimidine metabolism, amino acid metabolism, glycine, serine, threonine metabolism, and arginine and proline metabolism.

### 3.4. Effect of QFSS on Lung Metabolite Levels in Asthmatic Mice

**Figure 5:**
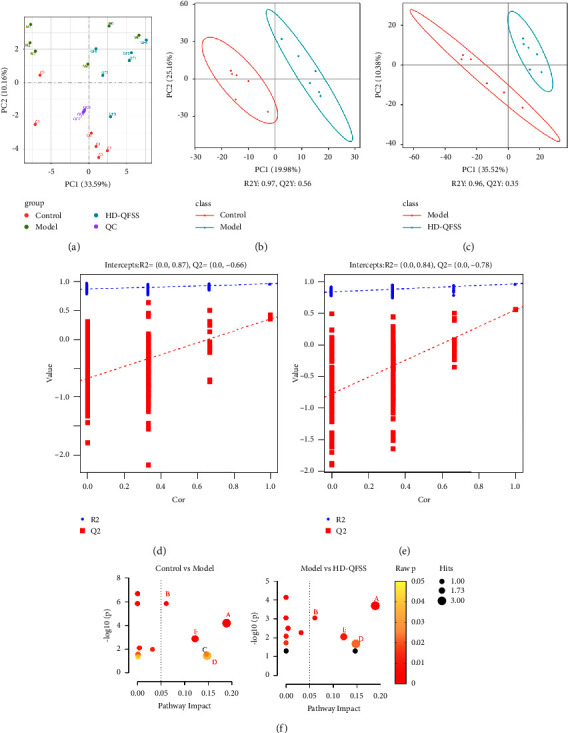
QFSS treatment regulated the lung metabolites in asthmatic mice. (a) Scores plots of PCA analysis among control, model, HD-QFSS group, and QC samples. (b, c) Scores plots of PLS-DA between control and model groups and between model and HD-QFSS groups. (d, e) Correlation coefficient among each group. (f) Bubble plot of pathway analysis among each group. Red bubbles indicated the common pathways. Black bubbles indicated the pathways with raw *P* ≥ 0.05. A: arginine and proline metabolism; B: arginine biosynthesis; C: starch and sucrose metabolism; D: pyrimidine metabolism; E: glycerophospholipid metabolism.

We further used untargeted metabolomic analysis to explore the effects of QFSS on pulmonary metabolites in asthmatic mice. The principal component analysis (PCA) score plot showed that the control group could be well differentiated from the model group, and the model group was well differentiated from the HD-QFSS group (Figure 5(a)). QC samples were clustered in PCA plots, indicating a good stability of the metabolomics analysis system [22]. The partial least squares discriminant analysis (PLS-DA) model was constructed to assess the explanatory power (*R*^2^) and predictive power (*Q*^2^) of the group and to identify the differential metabolites. Compared to the control group, *R*^2^*Y* = 0.97 and *Q*^2^*Y* = 0.56 in the model group (Figure 5(b)) and *R*^2^*Y* = 0.96 and *Q*^2^*Y* = 0.35 in the HD-QFSS group (Figure 5(c)). Besides, permutation tests showed that the PLS-DA model was stable and had a good predictive power (Figures 5(d) and 55(e)).

The following two criteria were used to screen for differential metabolites: *P* < 0.05 and VIP > 1.0 (control vs. model groups, or model vs. HD-QFSS groups). A total of 43 differential metabolites were screened (Table 3). Compared to the control group, metabolites such as methyl 10-undecenoate, L-fucose, and L-ornithine were significantly higher in the model group, and metabolites such as 1-oleoyl-sn-glycero-3-phosphocholine, 2-deoxyuridine, and 3-amino-4 methylpentanoic acid and other metabolites were significantly decreased. Compared to the model group, metabolites such as 2-(acetylamino)-3-[4-(acetylamino) phenyl] acrylic acid, D-raffinose, and LysoPC (15 : 1) were significantly higher in the HD-QFSS group, and metabolites such as methyl 10-undecenoate, PE (18 : 1/20 : 4), D-glucose6-phosphate, and other metabolites were significantly lower in the HD-QFSS group. In addition, we used MetaboAnalyst software to analyze the metabolic pathways of the 43 differential metabolites obtained (FC > 1.2 or FC < 0.8). Compared to the control group, the metabolic pathways that were altered in the model group mainly included arginine and proline metabolism, arginine biosynthesis, starch and sucrose metabolism, pyrimidine metabolism, and glycerophospholipid metabolism; while the metabolic pathways affected by QFSS mainly included arginine and proline metabolism, arginine biosynthesis, pyrimidine metabolism, and glycerophospholipid metabolism (Figure 5(f)). The common pathways between PICRUSt analysis of 16S rRNA sequencing and pathway analysis of untargeted metabolomics included arginine and proline metabolism and pyrimidine metabolism pathways, indicating that QFSS may regulate these metabolic pathways to treat asthma by affecting the gut microbiota. Therefore, these pathways were discussed in detail.

### 3.5. Spearmen's Analysis of the Correlations between the Results of 16S rRNA Sequencing, Therapeutic Effects, and Untargeted Metabolomics

**Figure 6:**
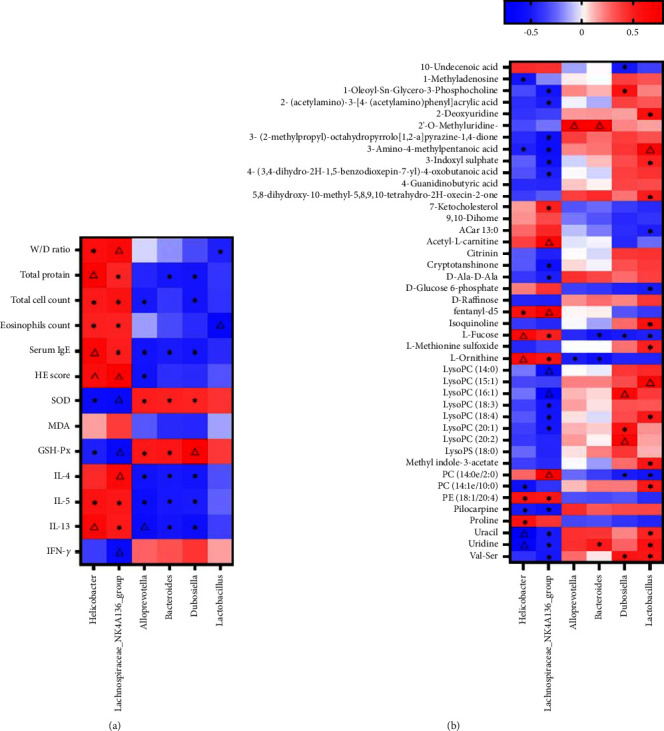
Spearmen's correlation analysis (heatmap). (a, b) Correlations between therapeutic indicators and gut microbiota (a) and between untargeted metabolomics and gut microbiota (b). Color coding scale indicates the correlation coefficient from heatmap, and the deeper red or blue indicates the higher absolute of the value. ^*∗*^*P* < 0.05; ^△^*P* < 0.01.

We further used Spearmen's analysis in order to study the relationship of differential gut microbiota with the therapeutic indicators and changed metabolites. As shown in Figure 6(a), *Lachnospiraceae_NK4A136_group*, *Helicobacter*, *Alloprevotella*, *Bacteroidota*, and *Dubosiella* exhibited significant correlations with most of the therapeutic indicators. Besides, *Lachnospiraceae_NK4A136_group*, *Helicobacter*, *Dubosiella,* and *Lactobacillus* exhibited significant correlations with many differential metabolites (Figure 6(b)).

## 4. Discussion

In the present study, we constructed an asthma model mouse using OVA. The results showed that the model group mice had reduced lung function and lung histopathological examination revealed a large number of inflammatory cell infiltrates in lung tissue. A previous study has shown that the W/D ratio of the lung was increased in asthmatic model mice [23]. The increase in lung W/D ratio could reflect the increase in lung permeability, and the lung permeability is impaired during the progression of asthma. In addition, the lung tissue W/D ratio of mice in the model group was increased, and the number of total BALF cells, eosinophils, and total protein were increased, indicating increased lung tissue permeability and increased serum IgE levels. These results were consistent with the pathological manifestations of asthma [23], suggesting that the model was successfully constructed. QFSS intervention significantly improved lung function, reduced lung tissue permeability, and downregulated serum IgE levels in asthmatic mice. In addition, we selected DXM as a positive control drug, which is a hormonal drug commonly used in the treatment of asthma [24]. The results showed that high-dose QFSS did not differ significantly from the DXM in improving lung function and reducing lung tissue permeability in asthmatic mice, and these results confirmed the therapeutic effects of QFSS in asthma.

Airway inflammation and oxidative stress are important pathological manifestations of asthma, and we next investigated the effects of QFSS on airway inflammation and oxidative stress in asthmatic mice. The results showed that the QFSS intervention reduced IL-4, IL-5, and IL-13 levels and increased IFN-*γ* levels in BALF of asthmatic mice. T helper cell (Th) 1/Th2 balance is important for maintaining the immune homeostasis of the body, and Th1/Th2 imbalance is an important mechanism for the development of airway inflammation in asthma. A study has found that there were reduced levels of Th1 in asthma, which reduces the levels of certain Th1-secreted cytokines including IFN-*γ*, and elevated levels of Th2, which can produce large amounts of IL-4, IL-5, and IL-13. These secreted cytokines can exacerbate the occurrence and progression of allergic reactions and promote Th2 differentiation, thereby inhibiting Th1 differentiation [25]. Decreasing the Th2-associated cytokines IL-4, IL-5, and IL-13, and increasing the Th1 cytokine IFN-*γ* may help to alleviate the inflammatory response in asthma. Our results also showed that QFSS increased SOD and GSH-Px activities and decreased MDA levels in the lung tissue of asthmatic mice, suggesting that QFSS inhibits oxidative stress in asthmatic mice. Oxidative stress is also an important pathological response in asthma as the accumulation of considerable amounts of reactive oxygen species (ROS) occur in the airways during the onset of asthma and these ROS exacerbate lipid peroxidation reactions and cause respiratory epithelial cell damage [26]. SOD and GSH-Px are important antioxidant enzymes that promote ROS scavenging. MDA is an end product of lipid peroxidation and its elevated level can reflect the severity of oxidative stress [27].

In this study, 16S rRNA high-throughput sequencing technology was used to study the effect of QFSS on the structure and composition of gut microbiota in asthmatic mice. Alpha diversity of gut microbiota refers to the diversity of microbiota within a specific region or ecosystem, and is a comprehensive indicator of the richness and homogeneity of the microbiota. Our results show no significant differences in the alpha diversity of gut microbiota in each group, indicating that the total numbers of OTUs were similar in each group. Therefore, we measured the beta diversity of gut microbiota in each group. The beta diversity of mouse gut microbiota was analyzed by PCoA and clustering analysis. The overall structure and composition of the gut microbiota of asthmatic mice changed significantly, QFSS could affect the beta diversity of the gut microbiota of asthmatic mice, and the beta diversity of the gut microbiota of asthmatic mice was more similar to the control group after QFSS intervention. The results of the analysis of the relative abundance of gut microbiota showed that QFSS could decrease the high levels of the F-to-B ratio caused by asthma. Changes in the F-to-B ratio are intimately associated with many diseases and metabolic disorders and inflammatory responses can be alleviated by decreasing the F-to-B ratio [28].

In addition, the relative abundance of *Lachnospiraceae_NK4A136_group* and *Helicobacter* was significantly increased and the relative abundance of *Lactobacillus*, *Alloprevotella*, *Bacteroidota*, and *Dubosiella* was significantly decreased in the mouse model of asthma; QFSS could significantly increase the relative abundance of *Lactobacillus* and *Dubosiella,* and decrease the relative abundance of *Lachnospiraceae_NK4A136_group* and *Helicobacter*. In a previous study, *Lachnospiraceae_NK4A136_group* was significantly and positively correlated with IgE and IL-33 [29]. *Helicobacter* is a Gram-negative spiral bacterium that is closely associated with many gastrointestinal diseases [30]. However, there is controversy over the effect of *Helicobacter* in asthma. Some studies have reported a degree of the protective effect of *Helicobacter* against asthma; however, other studies have pointed out that there is no negative association between *Helicobacter* and asthma [31]. Therefore, the interaction between *Helicobacter* and asthma requires further in-depth study. *Lactobacillus*, a natural microorganism with immunomodulatory abilities, has been shown to alleviate respiratory diseases such as asthma in several animal studies and clinical trials [32]. High-throughput sequencing studies of the gut microbiota of asthmatic patients have revealed a lower abundance of *Alloprevotella* in treated patients [33]. *Bacteroidota* in the gut metabolizes polysaccharides and oligosaccharides to provide nutrients and vitamins to the host and other gut microbiota. However, when *Bacteroides* colonize other sites, it has the potential to become opportunistic pathogens [34]. In a study on allergic asthmatic mice, it was found that *Faecalibacterium prausnitzii* may alleviate pathological changes in asthmatic mice by regulating the production of *Dubosiella* and short-chain fatty acids in gut microbiota [35]. At the same time, our correlation analysis showed that *Helicobacter* and *Lachnospiraceae NK4A136 groups* were positively correlated with the vast majority of asthma pathological indicators and proinflammatory factors, and negatively correlated with SOD, GSH-Px, and IFN-*γ*. In contrast, *Alloprevotella*, *Bacteroidota*, and *Dubosiella* behaved in contrast to the aforementioned microbiota, but failed to show statistical significance in eosinophil counts and IFN-*γ*. *Lactobacillus* showed a significant negative correlation only with the W/D ratio and eosinophil count. Notably, *the Lachnospiraceae NK4A136 group* showed an extremely wide range of significant associations, suggesting that we can focus on studying the deeper relationship between this group and asthma.

Untargeted metabolomics of lung homogenates showed that QFSS affects arginine and proline metabolism, arginine biosynthesis, glycerophospholipid metabolism, and pyrimidine metabolism in asthmatic mice. After correlating the differential metabolic pathways obtained from metabolomics with those deduced from 16s rRNA sequencing, arginine and proline metabolism, and pyrimidine metabolism metabolic pathways were identified as their common pathways, suggesting that QFSS may influence metabolic pathways through the regulation of gut microbiota, thus exerting the effect of asthma treatment.

### 4.1. Arginine and Proline Metabolism

In our study, we found that 4-guanidinobutyric acid levels decreased in asthmatic mice, while both L-ornithine and proline levels increased. The 4-guanidinobutyric acid levels increased and both L-ornithine and proline levels decreased significantly after treatment with QFSS. Both 4-guanidinobutyric acid and L-ornithine are downstream metabolites of arginine. Studies have shown that arginine metabolism plays an important role in asthma, which is related to nitric oxide (NO) metabolism in arginine biosynthesis, and high exhaled NO is one of the representative features in most asthmatic patients [36]. Coincidentally, L-ornithine is also one of the important metabolic intermediates in arginine biosynthesis and L-ornithine is also a precursor of several asthma-related metabolites, and its derivative proline, a precursor of collagen, is associated with airway remodeling [37], while the derivative spermine increases airway sensitivity to methacholine [38]. The 4-guanidinobutyric acid has been shown to have anti-*H. pylori* effects [39], which is consistent with the results of our correlation analysis. However, the results did not show sufficient statistical significance between 4-guanidinobutyric acid and gut microbiota. QFSS may affect 4-guanidinobutyric acid through other pathways, which still need to be further studied. Moreover, *Helicobacter* and *Lachnospiraceae NK4A136 groups* were positively correlated with L-ornithine and proline, while *Alloprevotella* and *Bacteroidota* were negatively correlated with L-ornithine.

### 4.2. Pyrimidine Metabolism

Our study found that lung levels of 2-deoxyuridine, uracil, and uridine were significantly decreased in mice with asthma models. All these metabolites were significantly elevated after QFSS treatment. Many experiments have demonstrated that uridine has anti-inflammatory effects and is able to suppress the classical characteristics of asthma airway inflammation [40, 41]. Uracil can be interconverted with 2-deoxyuridine or uridine. However, there are very few studies on uracil and 2-deoxyuridine in asthma and we failed to find valuable results. Hence, further studies are needed to explore the possible potential relationship. We found negative correlations between *Helicobacter* and *Lachnospiraceae NK4A136 group* and uracil and uridine, while positive correlations were present between *Lactobacillus* and uracil and uridine and 2-deoxyuridine. There was also a positive correlation between *Bacteroidota* and uridine.

## 5. Conclusion and Future Prospective

Recently, multiomics techniques are widely used in elucidating the mechanisms of Chinese herbal formulas. The use of multiomics techniques can better illuminate the multicomponents and multitargets of Chinese herbal formulas [42]. Our results showed that QFSS could ameliorate asthma in mice. The possible mechanism of QFSS on asthma may be associated with regulating gut microbiota and arginine and proline metabolism and pyrimidine metabolism. Our study may be useful for researchers to study the integrative mechanisms of Chinese herbal formulas based on modulating gut microbiota and metabolism.

In future, the fecal transplantation and the gut microbiota depletion model can be used to further validate the detailed mechanisms of QFSS on asthma based on modulating gut microbiota and host metabolism. Besides, our future study will deeply evaluate the effects of QFSS on the expression of relative metabolic enzymes, in order to deeply elucidate the metabolic regulatory mechanism of QFSS. Moreover, studies showed that the changes of metabolism in immune cells (such as macrophages, neutrophils, and lymphocytes) are important immune mechanism of asthma [43]. The effects of QFSS on metabolism in immune cells can also be studied in our future study.

There are also some limitations of our study. Only high-dose QFSS treatment mice were selected for the gut microbiota and metabolomics analysis in our study. Further study can be carried out to evaluate the effects of different dosages of QFSS on gut microbiota and metabolites in the asthmatic model mice.

## Figures and Tables

**Table 1:** Effects of QFSS on Penh in asthmatic mice.

Groups	Acetylcholine (mg/mL)				
	0	6.25	12.5	25	50
Control	44.49 ± 9.05	80.32 ± 14.44	109.25 ± 22.69	114.53 ± 20.22	125.57 ± 20.08
Model	44.91 ± 7.57	120.48 ± 21.12^##^	250.90 ± 51.18^##^	301.46 ± 40.86^##^	386.82 ± 58.01^##^
DXM	44.56 ± 9.48	91.22 ± 17.91^*∗∗*^	174.47 ± 48.94^*∗∗*^	227.94 ± 63.53^*∗∗*^	285.59 ± 48.34^*∗∗*^
LD-QFSS	42.52 ± 6.93	106.63 ± 20.38	204.75 ± 47.69	280.42 ± 34.08	342.63 ± 53.38
MD-QFSS	46.02 ± 6.93	103.58 ± 30.75	196.85 ± 45.00^*∗∗*^	267.87 ± 66.89^*∗∗*^	334.99 ± 56.72^*∗∗*^
HD-QFSS	44.66 ± 5.78	96.37 ± 22.13^*∗*^	182.55 ± 20.17^*∗∗*^	233.16 ± 45.00^*∗∗*^	296.94 ± 46.10^*∗∗*^

Control, model, DXM, LD-QFSS, MD-QFSS, and HD-QFSS groups (*n* = 10 per group). ^##^*P* < 0.01 compared with the control group; ^*∗*^*P* < 0.05 compared with the model group; ^*∗∗*^*P* < 0.01 compared with the model group.

**Table 2:** Effects of QFSS on SOD and GSH-Px activities and MDA level in lung tissue homogenate.

Group	SOD (U/mgprot)	MDA (nmol/mgprot)	GSH-Px (U/mgprot)
Control	59.14 ± 10.03	2.05 ± 0.53	21.44 ± 5.14
Model	26.83 ± 8.74^##^	4.56 ± 1.45^##^	6.95 ± 2.18^##^
DXM	43.29 ± 15.06^*∗∗*^	2.84 ± 1.11^*∗∗*^	13.99 ± 3.95^*∗∗*^
LD-QFSS	36.25 ± 13.61	3.66 ± 1.24	9.92 ± 3.15^*∗*^
MD-QFSS	38.88 ± 10.94^*∗*^	3.21 ± 1.10^*∗*^	11.50 ± 3.72^*∗∗*^
HD-QFSS	42.12 ± 13.13^*∗∗*^	3.06 ± 0.84^*∗*^	13.01 ± 4.85^*∗∗*^

Control, model, DXM, LD-QFSS, MD-QFSS, and HD-QFSS groups (*n* = 10 per group). ^##^*P* < 0.01 compared with the control group; ^*∗*^*P* < 0.05 compared with the model group; ^*∗∗*^*P* < 0.01 compared with the model group.

**Table 3:** Differential metabolites in asthmatic mice after the treatment of QFSS.

No.	Formula	RT (min)	m/z	Metabolites	VIP		FC		Trend		Pathway
					M vs. C	Q vs. M	M vs. C	Q vs. M	M vs. C	Q vs. M	
1	C_11_H_20_O_2_	12.77	183.14	Methyl 10-undecenoate	1.34	1.34	1.51	0.60	↑^##^	↓^*∗∗*^	
2	C_26_H_52_NO_7_P	15.01	520.34	1-Oleoyl-Sn-glycero-3-phosphocholine	2.30	1.01	0.50	1.46	↓^##^	↑^*∗*^	**e**
3	C_13_H_14_N_2_O_4_	8.29	263.10	2-(Acetylamino)-3-[4-(acetylamino)phenyl]acrylic acid	1.98	1.50	0.50	1.96	↓^#^	↑^*∗∗*^	
4	C_9_H_12_N_2_O_5_	1.61	227.07	2-Deoxyuridine	1.33	1.01	0.63	2.37	↓^##^	↑^*∗*^	**d**
5	C_11_H_18_N_2_O_2_	8.96	211.14	3-(2-Methylpropyl)-octahydropyrrolo[1,2-a]pyrazine-1,4-dione	2.51	1.40	0.41	1.89	↓^##^	↑^*∗*^	
6	C_6_H_13_NO_2_	1.37	132.10	3-Amino-4-methylpentanoic acid	2.14	1.66	0.56	1.82	↓^##^	↑^*∗∗*^	
7	C_13_H_14_O_5_	10.02	251.09	4-(3,4-Dihydro-2H-1,5-benzodioxepin-7-yl)-4-oxobutanoic acid	1.75	1.67	0.46	3.03	↓^#^	↑^*∗*^	
8	C_5_H_11_N_3_O_2_	1.41	146.09	4-Guanidinobutyric acid	2.45	1.67	0.24	3.40	↓^##^	↑^*∗*^	**a**
9	C_27_H_44_O_2_	14.05	401.34	7-Ketocholesterol	1.47	1.23	1.44	0.69	↑^#^	↓^*∗*^	
10	C_9_H_17_NO_4_	1.42	204.12	Acetyl-L-carnitine	1.30	1.35	1.28	0.72	↑^#^	↓^*∗*^	
11	C_13_H_14_O_5_	10.02	233.08	Citrinin	1.75	1.66	0.45	3.12	↓^#^	↑^*∗*^	
12	C_6_H_12_N_2_O_3_	1.40	161.09	D-Ala-D-Ala	2.33	1.21	0.37	1.89	↓^#^	↑^*∗*^	
13	C_6_H_13_O_9_P	1.20	259.02	D-Glucose6-phosphate	1.28	1.08	6.05	0.34	↑^#^	↓^*∗*^	**c**
14	C_18_H_32_O_16_	1.44	503.16	D-Raffinose	1.95	1.10	0.16	6.36	↓^##^	↑^*∗∗*^	
15	C_9_H_7_N	10.55	130.06	Isoquinoline	1.54	1.69	0.50	2.63	↓^#^	↑^*∗*^	
16	C_6_H_12_O_5_	1.40	165.08	L-Fucose	1.73	1.37	9.14	0.26	↑^##^	↓^*∗∗*^	
17	C_5_H_11_NO_3_S	1.32	166.05	L-Methionine sulfoxide	1.69	2.08	0.68	1.84	↓^##^	↑^*∗∗*^	
18	C_5_H_12_N_2_O_2_	1.33	131.08	L-Ornithine	1.81	1.82	8.10	0.53	↑^##^	↓^*∗*^	**a**, **b**
19	C_23_H_46_NO_7_P	14.32	480.31	LysoPC (15 : 1)	1.09	1.73	0.79	1.61	↓^#^	↑^*∗∗*^	**e**
20	C_24_H_48_NO_7_P	14.50	492.31	LysoPC (16 : 1)	2.05	1.54	0.56	1.76	↓^#^	↑^*∗*^	**e**
21	C_26_H_48_NO_7_P	14.40	516.31	LysoPC (18 : 3)	1.86	1.45	0.59	1.76	↓^#^	↑^*∗*^	**e**
22	C_26_H_46_NO_7_P	14.06	516.31	LysoPC (18 : 4)	1.52	1.83	0.62	2.30	↓^#^	↑^*∗∗*^	**e**
23	C_28_H_56_NO_7_P	15.53	608.39	LysoPC (20 : 1)	1.87	1.00	0.73	1.23	↓^#^	↑^*∗*^	**e**
24	C_28_H_54_NO_7_P	15.26	606.38	LysoPC (20 : 2)	2.32	1.25	0.62	1.40	↓^##^	↑^*∗∗*^	**e**
25	C_24_H_48_NO_9_P	14.82	524.30	LysoPS (18 : 0)	1.87	1.59	0.44	2.50	↓^#^	↑^*∗*^	
26	C_11_H_11_NO_2_	10.55	190.09	Methyl indole-3-acetate	1.50	1.56	0.52	2.45	↓^#^	↑^*∗*^	
27	C_20_H_40_NO_4_	12.97	358.29	O-acylcarnitine	1.58	1.67	1.67	0.51	↑^#^	↓^*∗*^	
28	C_32_H_64_NO_7_P	16.56	606.45	PC (14 : 1*e*/10 : 0)	1.19	2.04	0.76	1.73	↓^##^	↑^*∗∗*^	
29	C_43_H_76_NO_8_P	16.57	764.53	PE (18 : 1/20 : 4)	1.71	1.34	2.11	0.40	↑^##^	↓^*∗∗*^	**e**
30	C_11_H_16_N_2_O_2_	9.34	209.13	Pilocarpine	2.80	1.34	0.45	1.66	↓^##^	↑^*∗∗*^	
31	C_5_H_9_NO_2_	1.39	116.07	Proline	1.32	1.85	1.75	0.49	↑^#^	↓^*∗*^	**a**
32	C_4_H_4_N_2_O_2_	1.54	113.03	Uracil	1.95	1.36	0.56	2.06	↓^##^	↑^*∗∗*^	**d**
33	C_9_H_12_N_2_O_6_	1.62	243.06	Uridine	1.84	1.09	0.53	2.02	↓^##^	↑^*∗∗*^	**d**
34	C_8_H_16_N_2_O_4_	1.40	205.12	Val-ser	1.75	1.59	0.58	1.97	↓^#^	↑^*∗∗*^	
35	C_10_H_14_N_2_O_6_	1.40	259.09	2′-O-Methyluridine	1.94	1.45	0.29	2.15	↓^#^	↑	
36	C_19_H_20_O_3_	1.56	297.14	Cryptotanshinone	1.97	1.25	0.52	1.74	↓^#^	↑	
37	C_24_H_50_NO_7_P	14.47	496.34	PC (14 : 0*e*/2 : 0)	1.46	1.44	21.81	0.16	↑^#^	↓	
38	C_11_H_15_N_5_O_4_	1.40	282.12	1-Methyladenosine	1.57	1.58	0.65	1.59	↓	↑^*∗∗*^	
39	C_8_H_7_NO_4_S	6.56	212.00	3-Indoxyl sulphate	1.78	1.82	0.48	3.00	↓	↑^*∗∗*^	
40	C_18_H_34_O_4_	14.35	315.25	9,10-Dihome	2.00	1.67	1.90	0.48	↑	↓^*∗*^	
41	C_22_H_28_N_2_O	12.33	342.26	Fentanyl-d5	1.34	1.72	1.84	0.43	↑	↓^*∗*^	
42	C_22_H_46_NO_7_P	14.35	526.31	LysoPC (14 : 0)	1.44	1.55	0.74	1.60	↓	↑^*∗*^	**e**
43	C_10_H_14_O_4_	12.15	181.09	Modiolide G	1.20	1.24	0.65	1.91	↓	↑^*∗*^	

Control, model, and HD-QFSS groups (*n* = 6 per group). RT, retention time; VIP, variable importance of projection; FC, fold change; ↑: content increased; ↓: content decreased; vs., versus; C, control group; M, model group; Q, HD-QFSS group; PC, phosphatidylcholine; PE, phosphatidylethanolamine; PS, phosphatidylserine. ^#^*P* < 0.05 compared with the control group. ^##^*P* < 0.01 compared with the control group; ^*∗*^*P* < 0.05 compared with the model group; ^*∗∗*^*P* < 0.01 compared with the model group. a, arginine and proline metabolism; b, arginine biosynthesis; c, starch and sucrose metabolism; d, pyrimidine metabolism; e, glycerophospholipid metabolism. The bold text in “pathway” column indicates the pathway which the metabolite related with. a: arginine and proline metabolism; b: arginine biosynthesis; c: starch and sucrose metabolism; d: pyrimidine metabolism; e: glycerophospholipid metabolism.

## Data Availability

The data used to support the findings of this study are available from the corresponding author upon request.

## Supplementary Materials

Supplementary Materials The detailed information of reagents and detailed protocol for untargeted metabolomic analysis are shown in supplementary materials.
