# Supplementary material for: Study on the Mechanism of Qing-Fei-Shen-Shi Decoction on Asthma Based on Integrated 16S rRNA Sequencing and Untargeted Metabolomics
Source: Evid Based Complement Alternat Med. 2023 Feb 15;2023:1456844. doi: 10.1155/2023/1456844 (PMC9946754; doi:10.1155/2023/1456844)
Supplement: Supplementary Materials — The detailed information of reagents and detailed protocol for untargeted metabolomic analysis are shown in supplementary materials. [file 1456844.f1.docx]

**Detailed information of reagents**

Ovalbumin (OVA; cat: A800) was obtained from Solarbio Biotechnology Co., Ltd. (Beijing, China). Dexamethasone (DXM; cat: S17003) was obtained from Shanghai yuanye Biotechnology Co., Ltd. (Shanghai, China). Total Protein (cat: A045-4), superoxide dismutase (SOD; cat: A001-3-2), methane dicarboxylic aldehyde (MDA; cat: A003-1-2), glutathione peroxidase (GSH-Px; cat: A006-2-1) assay kits were obtained from Nanjing Jiancheng Biological Engineering Institute (Nanjing, China). Enzyme-linked immunosorbent assay (ELISA) kits of mouse interferon gamma (IFN-γ; cat: EK280HS), interleukin (IL)-4 (cat: EK204), IL-5 (cat: EK205), IL-10 (cat: EK210), IL-13 (cat: EK213), immunoglobulin E (IgE; cat: EK275) were purchased from Multi Science Biotechnology Co., Ltd. (Hangzhou, China).

**Detailed protocol for untargeted metabolomic analysis**

**Liquid chromatography–mass spectrometry (LC-MS) analysis**

Metabolic profiling was analyzed using Vanquish LC system (Thermo Fisher, Germany) coupled with a Q Exactive™ HF mass spectrometer (Thermo Fisher, Germany). A Hypesil GOLD chromatographic column (C18; 2.1 mm × 100 mm; 1.9 μm) was used for chromatography. The column temperature was maintained at 40℃. The mobile phase A was 0.1% formic acid and the mobile phase B was methanol. The flow rate was 0.2 mL/min and the injection volume was 2 μL. The gradient was set as follows: 2% B (0 min), 2% B (1.5 min), 100% B (12 min), 100% B (14 min), 2% B (14.1 min), and 2% B (17 min)

Mass spectrometry (MS) was used to detect the positive and negative ion modes using an electrospray ionization (ESI) source simultaneously. The ESI source settings were as follows: spray voltage (3.2 kV); sheath gas flow rate (40 arb); aux gas flow rate (10 arb); capillary temperature (320℃); polarity (positive, negative); scan range selected (100–1500 m/z); while MS/MS secondary scan was data-dependent scans., A QC was added after every six samples throughout the experiment to assess the stability of the experiment.

**Data processing and analysis**

The raw files obtained from MS were imported into Compound Discoverer 3.1 (CD3.1, Thermo Fisher) software for data pre-processing. First, the data were briefly screened using parameters, such as retention time and mass-to-charge ratio, and then the peaks were aligned based on the retention time deviation of 0.2 min and mass deviation (part per million, ppm) of 5 ppm to make the identification more accurate. Subsequently, the data were aligned based on the settings of 5 ppm, signal intensity deviation, signal-to-noise ratio, minimum signal intensity, and adduct ions for peak extraction, and the quantification of peak area was performed at the same time. The molecular formula was then predicted by molecular ion peaks and fragment ions, and compared to mzCloud, mzVault, and MassList databases, from which the metabolites were identified. Metabolites with a relative standard deviation (RSD) less than 30% in QC samples were then retained as the final identification result for the subsequent analysis (Dai et al., 2017). Principal component analysis (PCA) and partial least squares discriminant analysis (PLS-DA) were conducted using metaX software for multivariate statistical analyses. Differential metabolites were screened based on *P* < 0.05 and VIP >1 and with a fold change (FC) >1.25 or FC <0.80. Metabolic pathway enrichment analysis was performed for differential metabolites based on the MetaboAnalyst analytic platform (<https://www.metaboanalyst.ca/>).

Reference

Dai, W., Xie, D., Lu, M., Li, P., Lv, H., Yang, C., et al. (2017). Characterization of white tea metabolome: Comparison against green and black tea by a nontargeted metabolomics approach. Food. Res. Int. 96. doi: 10.1016/j.foodres.2017.03.028.
